# Diagnostic value of regional myocardial flow reserve measurements using Rubidium-82 PET: Disclosures none

**DOI:** 10.1007/s10554-022-02644-6

**Published:** 2022-08-08

**Authors:** Sabine S. Koenders, Jorn A. van Dalen, Pieter L. Jager, Mohamed Mouden, Cornelis H. Slump, Joris D. van Dijk

**Affiliations:** 1 Department of Nuclear Medicine, Isala Hospital, PO Box 10400, 8000 GK Zwolle, The Netherlands; 2 Department of Medical Physics, Isala Hospital, PO Box 10400, 8000 GK Zwolle, The Netherlands; 3 Department of Cardiology, Isala Hospital, PO Box 10400, 8000 GK Zwolle, The Netherlands; 4 Technical Medical Centre, University of Twente, Enschede, the Netherlands

## Abstract

**Purpose:**

Visual assessment of Rubidium (Rb-82) PET myocardial perfusion images is usually combined with global myocardial flow reserve (MFR) measurements. However, small regional blood flow deficits may go unnoticed. Our aim was to compare the diagnostic value of regional with global MFR in the detection of obstructive coronary artery disease (oCAD).

**Methods:**

We retrospectively included 1519 patients referred for rest and regadenoson-induced stress Rb-82 PET/CT without prior history of oCAD. MFR was determined globally, per vessel territory and per myocardial segment and compared using receiver-operating characteristic analysis. Vessel MFR was defined as the lowest MFR of the coronary territories and segmental MFR as the lowest MFR of the 17-segments. The primary endpoint was oCAD on invasive coronary angiography.

**Results:**

The 148 patients classified as having oCAD had a lower global MFR (median 1.9, interquartile range [1.5–2.4] vs. 2.4 [2.0–2.9]), lower vessel MFR (1.6 [1.2–2.1] vs. 2.2 [1.9–2.6]) and lower segmental MFR (1.3 [ 0.9–1.6] vs. 1.8 [1.5–2.2]) as compared to the non-oCAD patients (p < 0.001). The area under the curve for segmental MFR (0.81) was larger (p ≤ 0.005) than of global MFR (0.74) and vessel MFR (0.78).

**Conclusions:**

The use of regional MFR instead of global MFR is recommended as it improves the diagnostic value of Rb-82 PET in the detection of oCAD.

## Introduction

Myocardial perfusion imaging (MPI) using positron emission tomography (PET) has a high diagnostic value in the detection of myocardial ischemia and is growing in its use [1]. The addition of absolute myocardial flow reserve (MFR) measurements to the visual assessment of PET images has become part of the clinical routine and provides additional information about the extent and functional importance of possible stenoses [2–7]. For the visual assessment physicians usually assess the relative uptake in the different regions of the myocardium by using the 17 segment model to detect possible ischemia or infarctions. With oxygen-15-labelled water (O-15 H2O) PET it is already common practice to assess regional flow values in the evaluation of obstructive coronary artery disease (oCAD) [8]. However, with Rubidium-82 (Rb-82) PET flow values are often only assessed for the myocardium as a whole (global) and assessing regional flows are not specifically recommended by European guidelines [2, 9]. Small regional blood flow deficits may therefore go unnoticed when only looking at global flow values, potentially limiting the diagnostic value of Rb-82 PET. Hence, our aim was to compare the diagnostic value of regional MFR with global MFR measurements using Rb-82 PET in the detection of oCAD.

## Materials and methods

### Study population

We retrospectively included 1519 consecutive patients without prior history of oCAD referred for rest and regadenoson-induced stress Rb-82 PET/CT (GE Discovery 690, GE Healthcare) between May 2017 and February 2019. We routinely use this PET technique for all patients. Information about the patients history, demographics and risk factors were obtained by review of medical records and a questionnaire. As this study was retrospective approval by the medical ethics committee was not required according to Dutch law. Nevertheless, all patients provided written informed consent for the use of their data for research purposes.

### Patient preparation and data acquisition

All subjects were asked to refrain from caffeine containing substances for 24 h and to discontinue dipyridamole containing medication for 48 h before imaging. All patients underwent a MPI rest scan followed by a regadenoson-induced stress scan. Prior to the PET acquisition, a low-dose CT scan was acquired during free-breathing to provide an attenuation map of the chest. This scan was made using a 0.8 s rotation time, pitch of 0.97, collimation of 32 × 0.625 mm, tube voltage of 120 kV, and a tube current of 10 mA. Next, a fixed activity of 740 MBq Rb-82 was administered intravenously with a flow rate of 50 mL/min using a Strontium-82/Rb-82 generator (CardioGen-82, Bracco Diagnostics Inc.) immediately followed by a seven-minute PET list-mode acquisition. Ten minutes after the first activity bolus, we induced pharmacological stress by administering 400 µg (5 mL) of regadenoson over 10 s. After a 5 mL saline flush (NaCl 0.9%), we administered a second dose of 740 MBq Rb-82 followed by a 7 min stress PET acquisition.

### Image reconstruction

The low-dose CT scans were reconstructed using an iterative reconstruction method (70% adaptive statistical iterative reconstruction algorithm, ASIR) and a slice thickness of 5 mm. Attenuation correction was applied to all PET data. Next, we reconstructed dynamic PET data using 26 time frames (12 × 5 s, 6 × 10 s, 4 × 20 s and 4 × 40 s) with default settings as recommended by the manufacturer using 3D-ordered subset expectation maximization (OSEM) technique using 2 iterations and 24 subsets and a Gaussian post-smoothing filter of 12 mm while correcting for decay, attenuation, scatter and random coincidences and dead time effects. The voxel size was 3.3 × 3.3 × 3.3 mm^3^. Neither time-of-flight correction, nor a post-processing filter or resolution modelling was applied for the dynamic image reconstructions.

### Data analysis

We used Corridor4DM (Invia Medical Imaging Solutions, v2016.02.64) software to post-process the reconstructions. Myocardium contours were automatically detected in both rest and stress scans and manually realigned when necessary. Furthermore, a region of interest (ROI) was manually placed at the location of the mitral valve to estimate the activity in the blood pool. Next, the activity concentrations in the myocardium contour and ROI were measured in the 26 reconstructed time frames to calculate the time activity curves (TACs) for the left ventricle (LV), whole myocardium (global), left anterior descending (LAD), left circumflex (LCX) and right coronary (RCA) artery and for each of the 17-segments. The one-tissue compartment model of Lortie et al. [10] was used to calculate the myocardial blood flow (MBF) from the TACs using Corridor4DM. Rest MBF was calculated without rate-pressure product correction. Furthermore, myocardial flow reserve (MFR) was calculated as the ratio of stress MBF divided by rest MBF for the myocardium as a whole (further referred by global MFR), the three vascular territories and for all 17 segments. Vessel MFR was defined as the lowest flow reserve of LAD, LCX and RCA territories and segmental MFR as the lowest flow reserve of the 17 segments. All relative Rb-82 PET images were also visually assessed by two expert readers and classified as normal or as abnormal, where abnormal was defined as having a reversible and/or irreversible defect.

### Follow-up

Our primary endpoint was a diagnosis of oCAD, as the purpose of Rb-82 PET in clinical practice is to assess the extent and functional importance of stenosis in order to tailor treatment and hopefully prevent the occurrence of hard events in the future. Patients were classified as having oCAD if follow-up included either a conclusive invasive coronary angiography (ICA) for CAD as defined by a significant fractional flow reserve measurement (< 0.8) or > 70% stenosis in the LAD, LCX or RCA, or > 50% stenosis in the left main on ICA during follow-up [11]. Patients who did not underwent ICA during follow-up or patients who underwent ICA but were diagnosed as not having oCAD were classified as non-obstructive CAD (non-oCAD). In addition, a composition of oCAD and occurrence of all-cause mortality was used as a secondary outcome.

### Statistical analysis

Patient characteristics and continuous variables were expressed as mean ± standard deviation (SD) or median [interquartile range]. Statistical analysis was performed using IBM SPSS (IBM SPSS Statistics for Windows, Version 26.0. Armonk, NY: IBM Corp). To assess differences between patient characteristics the Student’s t-test, Mann–Whitney U test or χ2-test were performed. Receiver-operating characteristic (ROC) analyses were conducted to evaluate and compare the diagnostic value of global, vessel and segmental MFR by paired-analyses of the difference of the area under the curve (AUC). A sub-analyses was performed to assess the difference of the AUC on scans visually classified as normal or abnormal. The level of statistical significance was set to p < 0.05.

## Results

The median follow-up was 23 months [interquartile range: 18–27] with a minimal follow-up of 12 months. Of the 1519 included patients 148 were classified as having oCAD according to ICA. Most cases (72%) of oCAD occurred within 90 days after the PET scan. Of the remaining 1371 patients, 17 (1%) underwent ICA but were classified as non-oCAD. An additional 49 patients died during follow-up. Patients in the oCAD group (n = 148) and non-oCAD group (n = 1371) did not differ regarding weight, height, body mass index (BMI) and the risk factors smoking, hypertension, dyslipidaemia and family history (p ≥ 0.06) as shown in Table 1. Yet, patients in the oCAD group were older, suffered more often from diabetes, and were more often male (p ≤ 0.02).

**Table 1:** Baseline characteristics and outcomes of the patient population (n = 1519)

Characteristic	Obstructive CAD (n = 148)	Non-obstructive CAD (n = 1371)	p values
Age (years)	69 ± 9	66 ± 11	0.002
Male gender (%)	71	49	< 0.001
Weight (kg)	88 ± 17	89 ± 20	0.89
Height (cm)	175 ± 9	174 ± 10	0.25
BMI (kg/m^2^)	29 ± 5	29 ± 6	0.53
Current smoking (%)	12	13	0.83
Hypertension (%)	68	62	0.19
Dyslipidaemia (%)	50	42	0.06
Diabetes (%)	28	20	0.02
Family history (%)	45	52	0.11
Global rest MBF	1.0 [0.8–1.3]	1.0 [0.8–1.3]	0.44
Vessel rest MBF	0.9 [0.8–1.2]	1.0 [0.8–1.2]	0.41
Segmental rest MBF	0.7 [0.6-1.0]	0.8 [0.6-1.0]	0.21
Global stress MBF	1.9 [1.5–2.4]	2.5 [2.1-3.0]	< 0.001
Vessel stress MBF	1.6 [1.2–2.2]	2.3 [1.9–2.8]	< 0.001
Segmental stress MBF	1.3 [0.8–1.7]	1.9 [1.6–2.4]	< 0.001
Global MFR	1.9 [1.5–2.4]	2.4 [2.0-2.9]	< 0.001
Vessel MFR	1.6 [1.2–2.1]	2.2 [1.9–2.6]	< 0.001
Segmental MFR	1.3 [0.9–1.6]	1.8 [1.5–2.2]	< 0.001
Time to follow-up (months)	22 [19–27]	23 [18–27]	0.79
PCI during follow-up (%)	53%	NA	NA
CABG during follow-up (%)	41%	NA	NA
Time to confirmation obstructive CAD (weeks)	5.4 [3.7–17.6]	NA	NA
Data presented as mean ± SD, median [interquartile range] or percentage; *PCI* percutaneous coronary intervention; *CABG* coronary artery bypass graft			

**Fig. 1:**
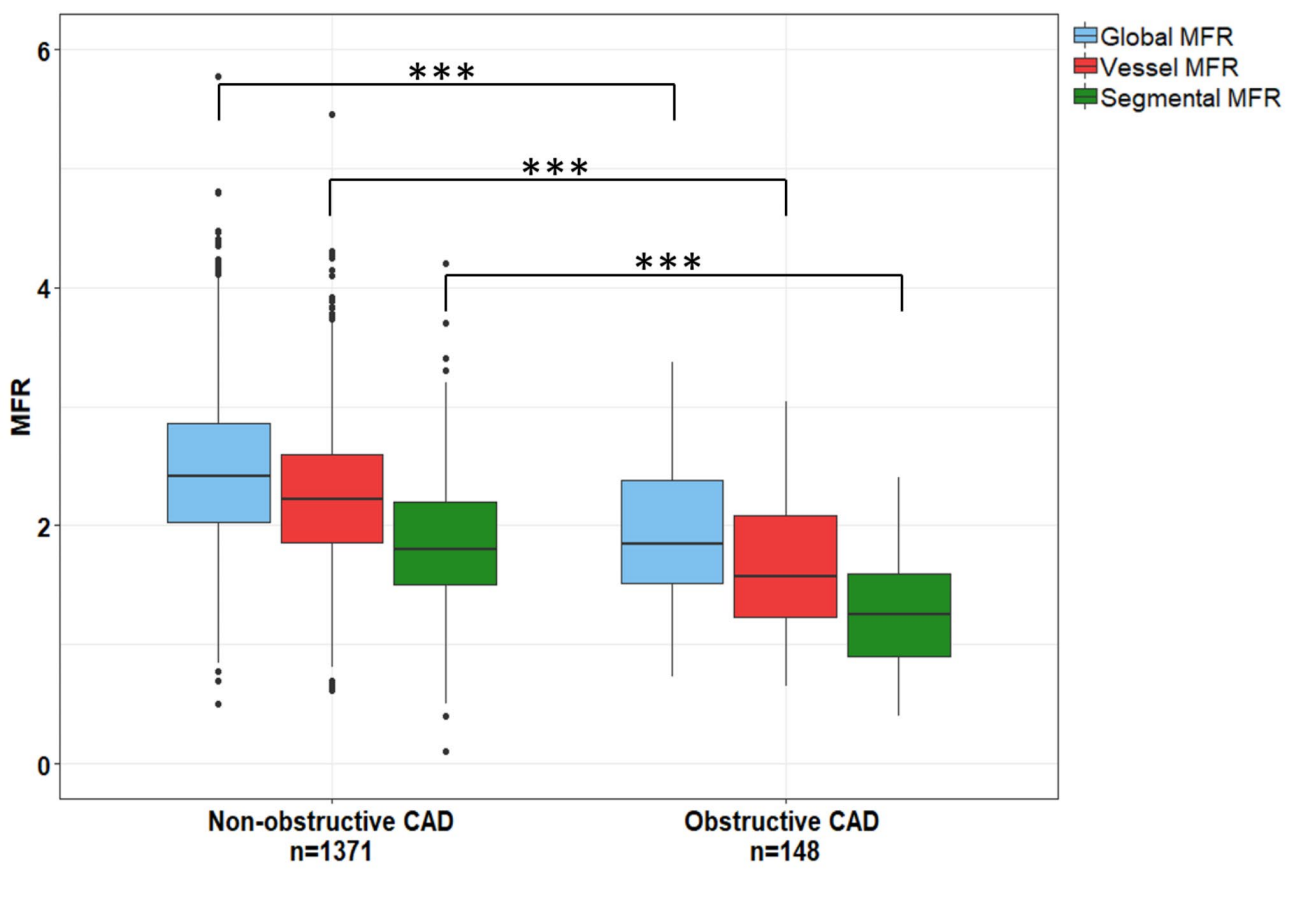
Boxplots showing the global, vessel and segmental MFR for all patients categorized as having non-obstructive CAD or obstructive CAD. Global, vessel and segmental flow values were significantly lower in the obstructive CAD group as compared to the non-obstructive CAD group *p < 0.05; **p < 0.01; ***p < 0.001

**Fig. 2:**
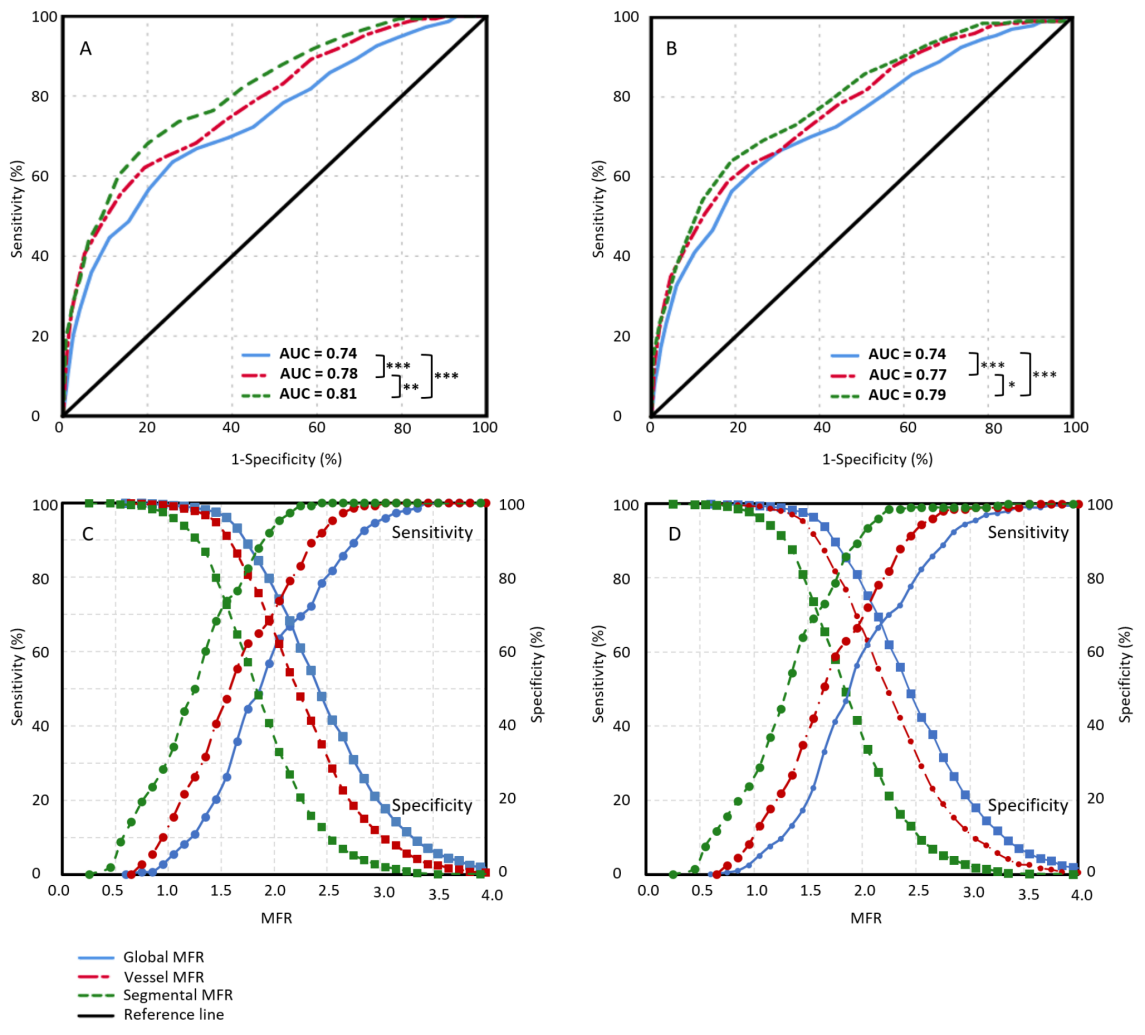
Receiver operating characteristic curves and sensitivity (round markers) and specificity (squared markers) pairs for detecting obstructive CAD (A & C) and obstructive CAD + all-cause mortality (B & D) for global, vessel and segmental MFR. The largest area under the curve was found for segmental MFR. *p < 0.05; **p < 0.01; ***p < 0.001

**Fig. 3:**
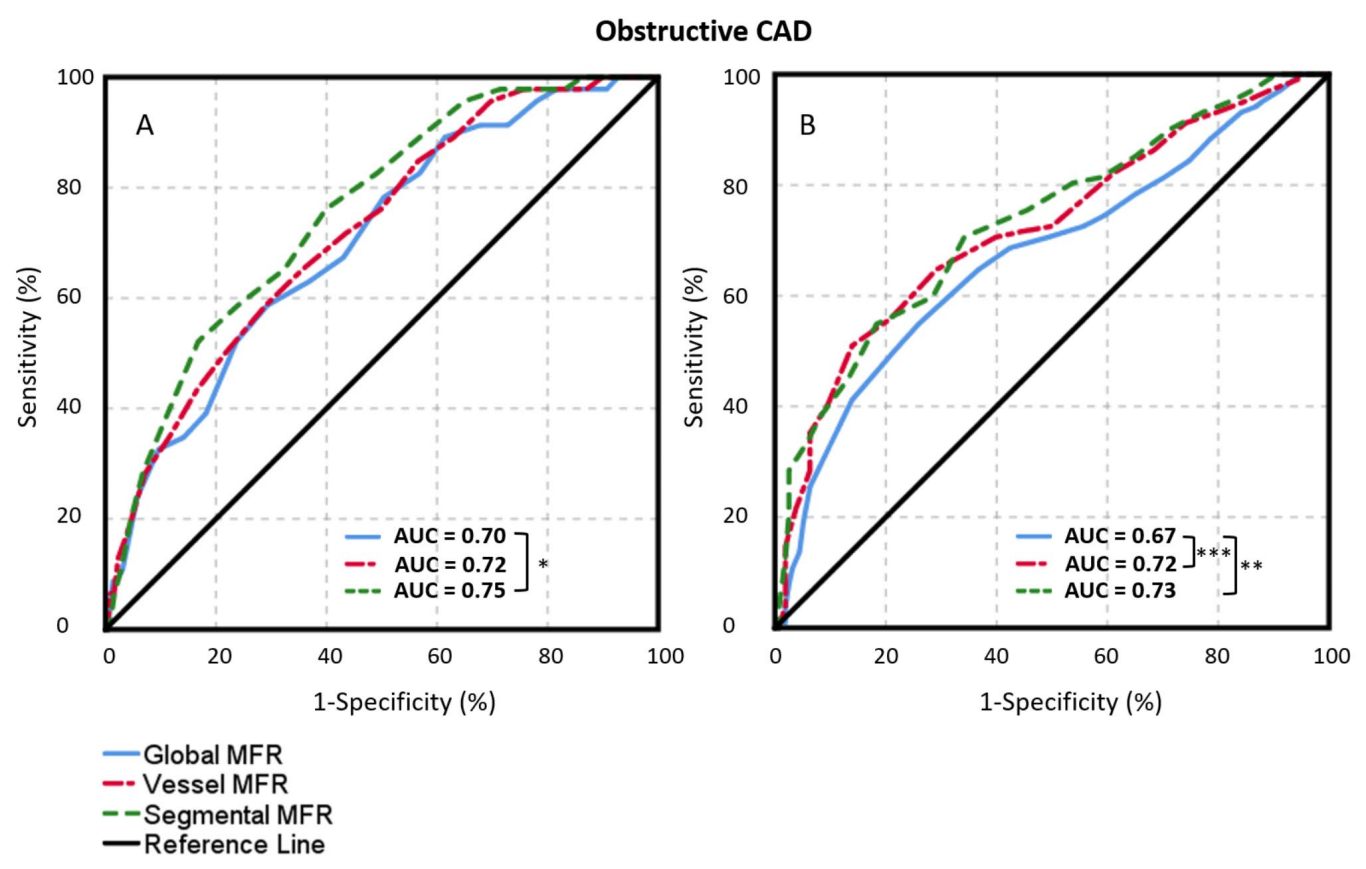
Receiver operating characteristic curves for detecting obstructive CAD, in scans classified as normal (A, n = 1259) and abnormal (B, n = 260) by visual assessment of expert readers, for global, vessel and segmental MFR. The largest area under the curve was found for segmental MFR. *p < 0.05; **p < 0.01; ***p < 0.001

The 148 patients classified as having oCAD had a lower global MFR (median 1.9 interquartile range [1.5–2.4] vs. 2.4 [2.0–2.9]), vessel MFR (1.6 [1.2–2.1] vs. 2.2 [1.9–2.6]) and segmental MFR (1.3 [ 0.9–1.6] vs. 1.8 [1.5–2.2]) in comparison to the non-oCAD patients, respectively (p < 0.001), as shown in Fig. 1. ROC analysis for oCAD showed that the AUC of segmental MFR (0.81) was significantly larger (p ≤ 0.005) than the AUC of global MFR (0.74) and vessel MFR (0.78), as shown in Fig. 2 A. To achieve the same sensitivity and specificity as for global MFR, the cut-off value for vessel and segmental MFR is lower as compared to global MFR, as shown in Fig. 2 C. Moreover, the trade-off between the sensitivity and specificity is dependent of the chosen cut-off value. After classification of all relative Rb-82 PET scans into normal (n = 1259) or abnormal (n = 260), the AUC of segmental MFR (0.75 and 0.73) was larger (p ≤ 0.047) than the AUC of global MFR (0.70 and 0.67), respectively, as shown in Fig. 3.

**Fig. 4:**
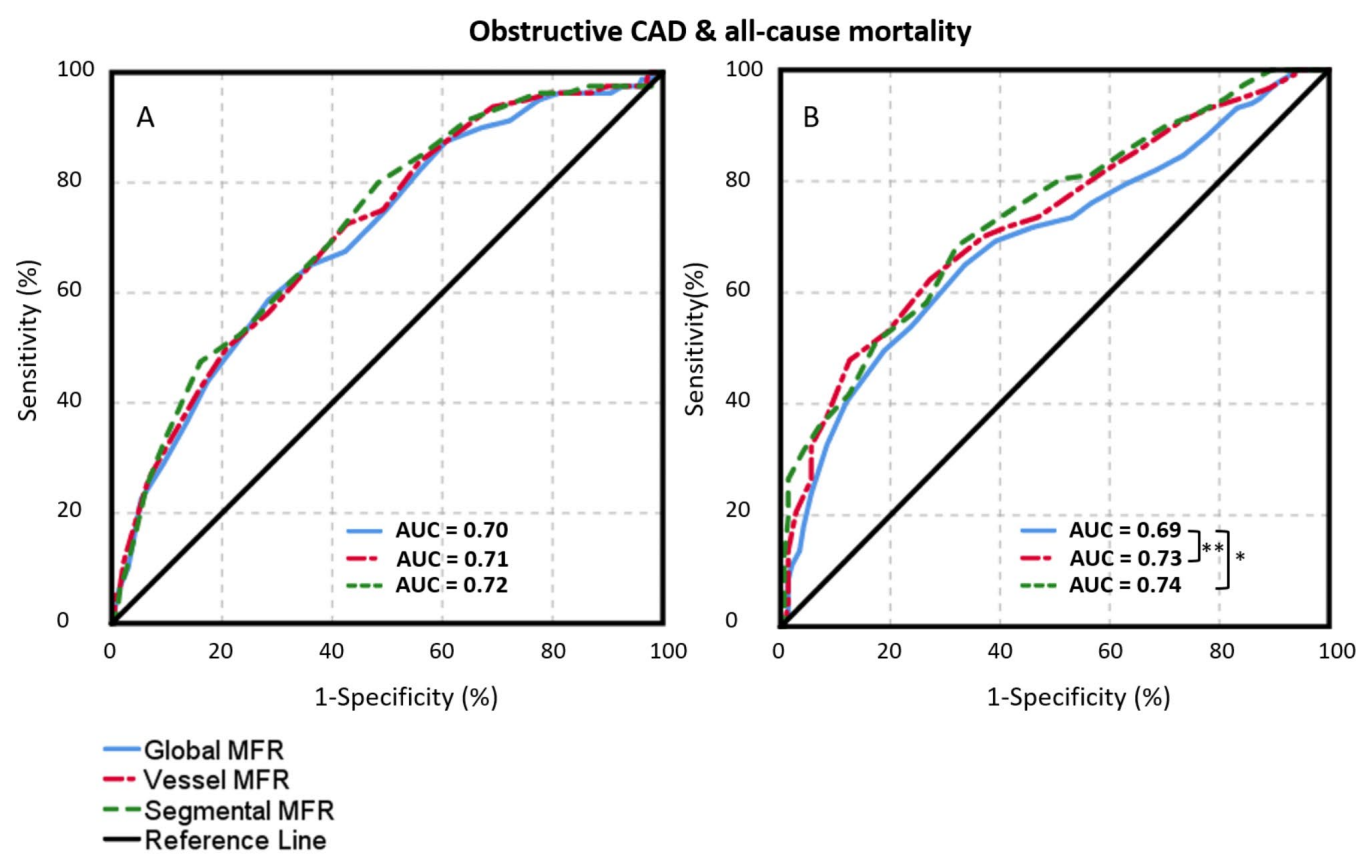
Receiver operating characteristic curves for detecting obstructive CAD + all-cause mortality, in scans classified as normal (A n = 1259) and abnormal (B, n = 260) by visual assessment of expert readers, for global, vessel and segmental MFR. The largest area under the curve was found for segmental MFR. *p < 0.05; **p < 0.01; ***p < 0.001

**Fig. 5:**
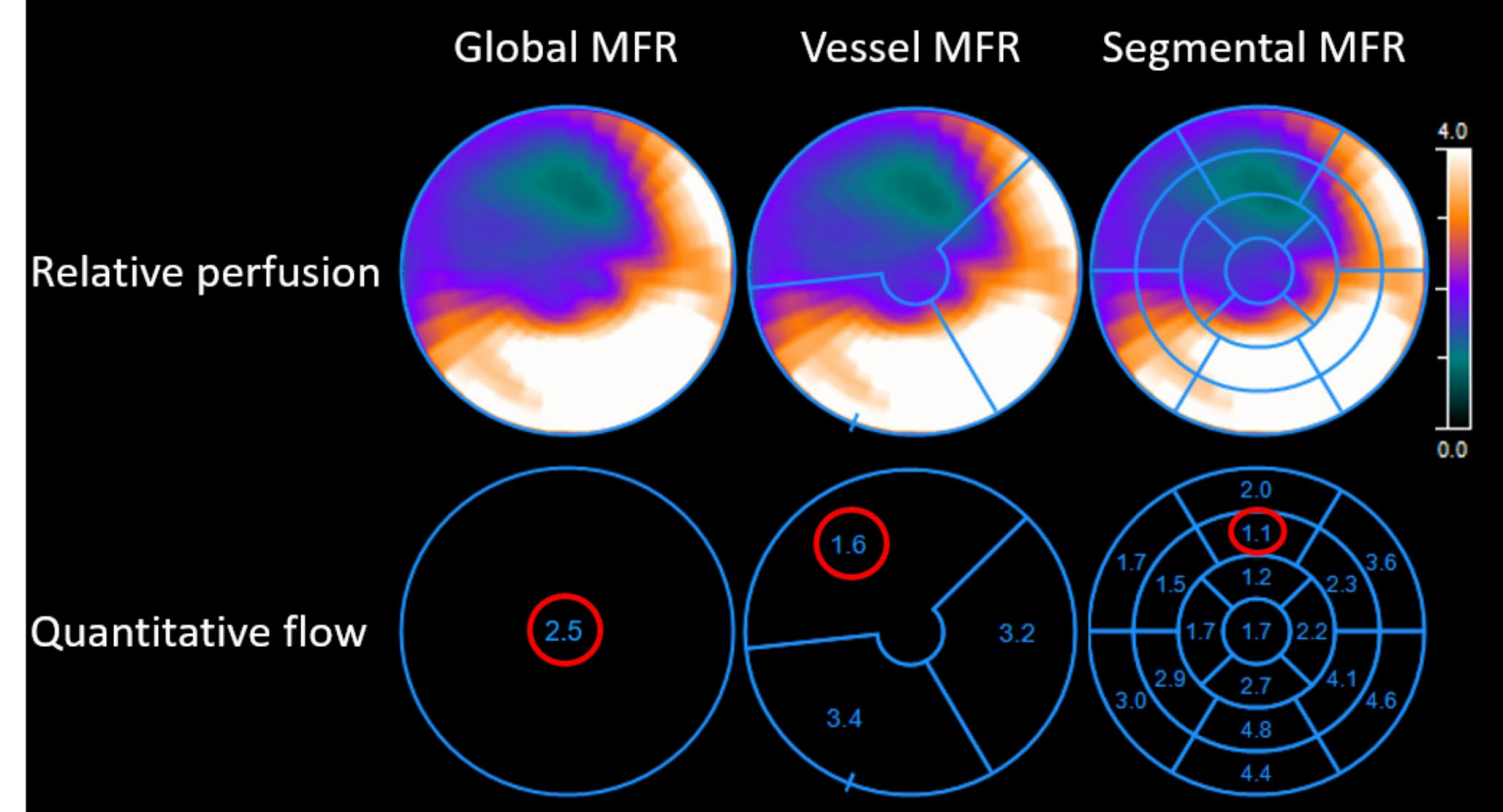
Example of a 65-year old male patient with a BMI of 33 kg/m^2^ who suffered from chest pain without prior history of CAD. According to the commonly used myocardial flow reserve (MFR) cut-off values [5] this patient would be classified as low risk (MFR > 2.0) for CAD according to the global MFR which is 2.5. However, this patient would be reclassified to intermediate risk (MFR 1.5-2.0) according to the vessel MFR of 1.6 for the LAD territory and reclassified as high risk (MFR < 1.5) according to the segmental MFR of 1.1. During invasive coronary angiography, single vessel disease of the proximal LAD with a subtotal stenosis was observed which was followed by PCI

When looking at the second endpoint, a composite of oCAD and all-cause mortality, segmental MFR also showed a higher AUC (0.79) as compared to global (0.74) and vessel MFR (0.77) (p ≤ 0.04) (Fig. 2B). After classification of all relative Rb-82 PET scans into normal or abnormal the AUC of segmental MFR (0.74) was larger in the abnormal Rb-82 PET scans as compared to global (0.69) MFR (p = 0.03), as shown in Fig. 4B. However, for the visually normal scans, the AUC of global, vessel and segmental MFR did not differ (p > 0.3). A case example demonstrating the higher diagnostic value of segmental MFR as compared to global MFR is shown in Fig. 5.

## Discussion

In this study we compared the diagnostic value of regional with global MFR measurements in Rb-82 PET using oCAD as primary endpoint. The patients classified as having oCAD had a lower global MFR, lower vessel MFR and lower segmental MFR as compared to the non-oCAD patients (p < 0.001). We showed that segmental MFR resulted in an improved detection of oCAD as compared to global MFR independent of the visual assessment.

Several study groups investigated the value of blood flow measurements with PET in the detection of oCAD, but none compared regional to global MFR measurement using Rb-82 PET. Mc Ardle et al. performed a meta-analysis of the diagnostic accuracy of relative Rb-82 PET perfusion imaging in the diagnosis and prognosis of patients with known or suspected oCAD [1]. They pooled the data from 15 studies resulting in a ROC curve with an AUC of 0.95. Our AUC of segmental MFR and global MFR of respectively 0.81 and 0.74 are relatively low as compared to the their reported AUC of 0.95. However, we only used quantitative flow measurements in computing the ROC curves, whereas Mc Ardle et al. assessed relative Rb-82 PET MPI. Ziadi et al. reported the added value of regional MFR in patients with known or suspected oCAD as a sub-analysis [3]. They found an increased major adverse cardiac event rate for patients with a normal global MFR but abnormal regional MFR in one of the vascular territories as compared to patients with normal MFR in all vascular territories. As it is well known that MFR provides valuable additional information to relative perfusion imaging in Rb-82 PET [2–7], the combination of visual assessment and quantitative flow measurements using Rb-82 PET would likely improve our AUCs. In addition, the study of Fiechter et al. determined the added value of MFR to relative MPI using nitrogen-13 ammonia PET for patients with suspected oCAD [12]. For the combined interpretation of relative images and MFR, patients with abnormal relative perfusion MPI were classified as abnormal regardless of MFR. Patients with normal relative MPI findings but abnormal MFR were reclassified from normal to abnormal. They found that the accuracy in the detection of oCAD improved from 79 to 89% after adding MFR to the visual assessment. However, they did not account for the added value of high MFR values in patients with visual abnormal scans as shown by Murthy et al. [2]. Moreover, with O-15 H2O PET the use of regional MFR is already part of the clinical routine and assessed in voxels presented in the 17-segments model of the myocardium [8].

This study had several limitations that should be recognized. First, our primary endpoint, oCAD, was based on epicardial stenosis visible using ICA. In our study, patients without oCAD but with a decreased MFR due to microvascular disease (MVD) were categorized as non-oCAD which reduces the accuracy of MFR in detecting oCAD [13]. If we used CAD instead of oCAD on ICA as endpoint, these patients would probably be correctly diagnosed using MFR, possibly resulting in improved ROC curves but our conclusion would likely not change. Despite our findings that regional MFR improves the diagnostic value of Rb-82 PET as compared to global MFR, there still might be a role for global MFR: it may be used to identify MVD [14, 15]. Yet low global MFR values are generally due to low regional MFR values. Hence regional MFR might be suitable to identify coronary microvascular dysfunction as well.

Second, we did not assess the correlation between regional MFR and the affected coronary territories identified by ICA. Although this could be of interest, there is a wide variability in coronary anatomy making the comparison of the affected coronaries based on ICA with the commonly used 17 myocardial segments frequently inaccurate [16].

Third, as our study was retrospective, a referral bias may have been introduced as referral for ICA could have been influenced by clinical information and a visual positive Rb-82 PET scan for ischemia or a myocardial infarction, or by a low global MFR. To limit this influence, we choose oCAD as primary endpoint including a median follow-up of 23 months to limit this effect. Furthermore, we expect that correction for this bias would result in regional MFR to be of even greater value as compared to global MFR in the detection of oCAD as the segmental MFR can already be reduced while the global MFR is within a normal range. Therefore, a perfusion deficit can be detected in an earlier stage using the segmental MFR as compared to the global MFR.

Next, we only assessed the diagnostic value of MFR using standardized acquisition and reconstruction parameters and not of stress MBF. There are several studies that report stress MBF being superior to MFR in risk stratification [17–19], while others report MFR being superior to stress MBF [2, 3, 5, 20, 21]. Although these studies are conflicting, MFR is less affected by technical variations such as reconstruction settings including temporal sampling, kinetic modelling and the software being used as compared to MBF [22–25]. Therefore, we only focused on MFR. Nevertheless, different technical settings might still influence the ROC curves using MFR. However, we expect that regional MFR still outperforms global MFR as long as acquisition, reconstruction parameters and processing software are standardized.

Finally, we did not assess the performance of qualitative perfusion images combined with quantitative flow values as this was outside the scope of this study. Yet, it would be interesting to study the value of MFR in addition to visual assessment, as this would be in line with current practice. However, such a study is not straight forward as different assumptions and thereby choices need to be made. First, the chosen cut-off value is dependent on the desired sensitivity and specificity. Second, in clinical practice two cut-off values are commonly used; a global MFR < 1.5 associated with a high risk on oCAD and a global MFR > 2.0 associated with a low risk oCAD [5], making the interpretation of sensitivity and specificity misleading.

## New knowledge gained

In this study, we showed that the use of regional MFR improves the diagnostic value of Rb-82 PET as compared to global MFR. It may lead to a change in risk classification. Hence, routine integration of segmental MFR rather than global MFR in combination with visual assessment of relative MPI scans seems promising when reporting Rb-82 PET images. In addition, the use of regional MFR could improve risk stratification in the detection of oCAD. A reason for the worse performance of global MFR in comparison to regional MFR likely is the compensation of poorly perfused parts by well perfused parts, possibly leading to under diagnosis of significant oCAD. Consequently, altered segmental cut-off values need to be applied to distinguish patients with oCAD from non-oCAD patients, as compared to global MFR cut-off values. Future studies will have to indicate which segmental MFR cut-off values are most suitable for this purpose.

## Conclusions

The diagnostic value of quantitative Rb-82 PET improved when using regional instead of global myocardial flow reserve in the detection of obstructive CAD. We therefore recommend to use the segmental flow reserve values in combination with visual assessment of Rb-82 PET scans.

## Abbreviations

AUC: Area under the curve.
CAD: Coronary artery disease.
ICA: Invasive coronary angiography.
LAD: Left anterior descending.
LCX: Left circumflex.
LV: Left ventricle.
MBF: Myocardial blood flow.
MFR: Myocardial flow reserve.
MPI: Myocardial perfusion imaging.
O-15: Oxygen-15.
PET: Positron emission tomography.
Rb-82: Rubidium-82.
RCA: Right coronary artery.
ROC: Receiver operating characteristic.
TAC: Time activity curve.
